# An appraisal of Indonesia’s immense peat carbon stock using national peatland maps: uncertainties and potential losses from conversion

**DOI:** 10.1186/s13021-017-0080-2

**Published:** 2017-05-19

**Authors:** Matthew Warren, Kristell Hergoualc’h, J. Boone Kauffman, Daniel Murdiyarso, Randall Kolka

**Affiliations:** 1 USDA Forest Service, Northern Research Station, 271 Mast Rd., Durham, NH 03824 USA; 2 Center for International Forestry Research, CIFOR c/o Centro Internacional de la Papa (CIP), Av. La Molina 1895, La Molina Apdo postal 1558, 15024 Lima, Peru; 3 Center for International Forestry Research, Jl. CIFOR, Situgede, Bogor, 16115 Indonesia; 4 Department of Fisheries and Wildlife, Oregon State University, 104 Nash Hall, Corvallis, OR 97331 USA; 5 Department of Geophysics and Meteorology, Bogor Agricultural University, Kampus Darmaga, Bogor, 16680 Indonesia; 6 USDA Forest Service, Northern Research Station, 1831 Hwy 169 East, Grand Rapids, MN 55744 USA

**Keywords:** Tropical peatlands, Wetlands, Climate change mitigation, Carbon pools, Carbon emissions, Peat degradation

## Abstract

**Background:**

A large proportion of the world’s tropical peatlands occur in Indonesia where rapid conversion and associated losses of carbon, biodiversity and ecosystem services have brought peatland management to the forefront of Indonesia’s climate mitigation efforts. We evaluated peat volume from two commonly referenced maps of peat distribution and depth published by Wetlands International (WI) and the Indonesian Ministry of Agriculture (MoA), and used regionally specific values of carbon density to calculate carbon stocks.

**Results:**

Peatland extent and volume published in the MoA maps are lower than those in the WI maps, resulting in lower estimates of carbon storage. We estimate Indonesia’s total peat carbon store to be within 13.6 GtC (the low MoA map estimate) and 40.5 GtC (the high WI map estimate) with a best estimate of 28.1 GtC: the midpoint of medium carbon stock estimates derived from WI (30.8 GtC) and MoA (25.3 GtC) maps. This estimate is about half of previous assessments which used an assumed average value of peat thickness for all Indonesian peatlands, and revises the current global tropical peat carbon pool to 75 GtC. Yet, these results do not diminish the significance of Indonesia’s peatlands, which store an estimated 30% more carbon than the biomass of all Indonesian forests. The largest discrepancy between maps is for the Papua province, which accounts for 62–71% of the overall differences in peat area, volume and carbon storage. According to the MoA map, 80% of Indonesian peatlands are <300 cm thick and thus vulnerable to conversion outside of protected areas according to environmental regulations. The carbon contained in these shallower peatlands is conservatively estimated to be 10.6 GtC, equivalent to 42% of Indonesia’s total peat carbon and about 12 years of global emissions from land use change at current rates.

**Conclusions:**

Considering the high uncertainties in peatland extent, volume and carbon storage revealed in this assessment of current maps, a systematic revision of Indonesia’s peat maps to produce a single geospatial reference that is universally accepted would improve national peat carbon storage estimates and greatly benefit carbon cycle research, land use management and spatial planning.

**Electronic supplementary material:**

The online version of this article (doi:10.1186/s13021-017-0080-2) contains supplementary material, which is available to authorized users.

## Background

Tropical peatlands are known to be globally significant deposits of terrestrial organic carbon with estimates ranging from 50 [1] to 105 GtC [2, 3]; equivalent to about 15% of carbon stored in peat globally. Peatlands occur throughout the tropics, covering between 33.4 and 57.8 Mha. This maximum estimate includes the 13.6 Mha of additional peatland that was recently reported for the Central Congo Basin [2, 3]. Southeast Asia contains the largest proportion of tropical peatland with an estimated 41% of tropical peatland area and 65% of the tropical peat C store (105 GtC) [2, 3]. Indonesia alone contains approximately 36% of the world’s tropical peatlands with frequently cited estimates of about 21 Mha (RePPProt [4], cited in Page et al. [2, 5–7]). Peatlands in Southeast Asia have formed over thousands of years with the oldest initiating around 20,000 years ago in the upper Kapuas Basin of West Kalimantan, Indonesia [8]. However, most peatlands in Southeast Asia are less than 7000 years old [9]. Forested ombrotrophic peat swamps have developed in landscapes where abundant precipitation and low relief produce high water tables and frequent inundation. These conditions are characteristic of the wide, low lying interfluves on the coastal plains of Sumatra, Borneo and Papua. Saturated peat soils are anoxic and nutrient poor, which impedes full decomposition of forest litter. Consequently, small amounts of residual organic matter accumulate and cause accretion of the peat layer [9–11].

Peat swamps in Sumatra and Kalimantan (Indonesian Borneo) have been large and persistent carbon sinks from around 15,000 years ago to present, sequestering between 0.5 and 1.5 Mg C/ha^1^ year^−1^ in peat [9, 12]. Although peat accumulation rates are only about 0.2–2.0 mm year^−1^, deposits up to several meters thick have formed over millennia, resulting in dense soil organic carbon pools that are stabilized by permanent anoxic conditions in the saturated peat profile [9, 12]. The resulting peat carbon stores are often in excess of 1000 Mg C/ha^1^ with values over 7500 Mg C/ha^1^ reported for exceptionally thick (>12 m) peat layers [13–15]. These carbon stores per area are immense compared to the average amount of carbon in the above-ground biomass of mature tropical rainforest, typically 130–240 Mg C/ha^1^ [16].

In addition to carbon sequestration, Indonesian peat swamp forests supply many ecological benefits to large coastal populations living in and around peatland landscapes. Peat swamp forests have been historical sources of timber and non-timber forest products including food, fiber, latex, medicine and materials for household goods. Peat swamps regulate hydrology by mitigating floods during wet seasons and intense rainfall events, and maintain base flows in streams and rivers during dry periods by slowly releasing stored water. Peat forests are also high in biodiversity and are critical habitat for many rare and endangered species including Sumatran tigers, orangutans, gibbons, and leopards [17–20]. Despite these values, Indonesian peat swamp forests are being deforested, drained and converted at unprecedented rates [21–23]. For example, Miettinen et al. [24] reported a 41.3% loss in peat swamp forest cover in Sumatra, and a 24.8% loss for the whole island of Borneo from 2000 to 2010. Trends in peatland conversion have continued with only 29% of peatlands remaining forested in Peninsular Malaysia, Borneo and Sumatra in 2015 [25]. Deforested peatlands are generally converted to industrial and smallholder plantations including oil palm (*Elaeis guineensis*) and acacia (*Acacia* spp.) for pulp and paper production. Miettinen et al. [25] estimated about 23,000 km^2^ of peat swamp forest were converted into industrial plantations by 2010 throughout Sumatra and the Kalimantan provinces of Indonesian Borneo.

Peat swamp conversion requires extensive drainage, and fire is often used to open land and remove undesired biomass to prepare for planting. Both of these activities cause large-scale greenhouse gas (GHG) emissions to the atmosphere [10, 26] which is the cause of major international concern. Drainage releases aerobic microbes from physiologically constraining anoxic conditions, resulting in rapid decomposition and heterotrophic CO_2_ production [27]. Decomposition may be further accelerated by additions of chemical fertilizers [28–30]. Net peat CO_2_ emissions are estimated to be 296 and 125 Mg CO_2_–C/ha^1^ for oil palm and *Acacia* plantations, over respective rotation periods of 25 and 6 years [31]. The IPCC guidelines [31] suggest an additional 88 Mg C/ha^1^ lost to the atmosphere from each land-clearing peat fire. Total (from peat and vegetation) carbon emissions from peat forest conversion to oil palm plantation are estimated to be between 350 and 487 Mg C/ha^1^ over a 25 year crop rotation [10, 31, 32]. These estimates are conservative since they exclude on-site non-CO_2_ GHG emissions, including CH_4_ emissions from drainage ditches, and N_2_O emissions from peat decomposition and application of nitrogenous fertilizers [31]. Per unit area, GHG emissions from tropical peatland conversion are higher than those from any other land use, land use change and forestry (LULUCF) activity. As a result, fire and peat emissions are Indonesia’s largest source of GHG (about 38% of total national emissions) and place Indonesia among the top five GHG emitting countries [33, 34]. Indonesia’s climate change mitigation efforts are therefore oriented towards avoiding GHG emissions from peatlands through conservation and restoration activities (e.g. REDD+).

From a national standpoint, quantifying Indonesia’s peatland carbon assets and associated uncertainties is critical for carbon valuation on emerging compliance and voluntary markets. Improving estimates of Indonesia’s peatland carbon store also has global implications: they are currently estimated to comprise 64% of the total tropical peat carbon pool [2]. There are several estimates of Indonesia’s peatland carbon store either regionally or nationwide (see Wahyunto et al. [2, 5–7, 13, 35]). However, only Wahyunto et al. [5–7] provide a national carbon storage estimate based on maps of peat distribution, thickness, and carbon density for Sumatra, Kalimantan and Papua islands. Here we revisit Indonesian peat carbon stores and their uncertainty using two current maps of peat area and thickness to calculate peat volume, and geographically specific peat carbon densities to calculate carbon mass.

### Indonesia peat map sources

We considered the two commonly used geospatial references for the extent and depth of Indonesian peatlands: (1) Maps of Indonesian peat distribution and carbon content published by Wetlands International and Wildlife Habitat Canada (hereafter referred to as WI maps; [5–7]) and (2) The Indonesia peatlands map published by the Center for Research and Development of Agricultural Land Resources, Agricultural Research and Development Agency, Indonesia Ministry of Agriculture (hereafter referred to as the MoA map; [36]).

The WI maps are widely used for research, and are reference data layers of peatland distribution in recent analyses of land-use change [22–25, 37–39], fire emissions from peatlands [26, 40], land cover mapping [23, 39], and geological history of peat formation [35]. These maps were produced using source data from the Land Resource Evaluation and Planning Project (LREP), implemented by several Indonesian government agencies from 1985 to 1990; secondary field data obtained from the Bogor Agriculture and Land Research Center; source data from Land Form Classification Maps produced by the Regional Planning Program for Transmigration [40], and analysis of Landsat satellite imagery acquired in 1990 and 2002 [5–7]. The WI maps estimate the total peatland area of Indonesia to be 20,949,043 ha.

The MoA map is the official government map of peatlands in Indonesia. It is used by government agencies as a reference for spatial planning, land management decisions and estimates of peat land use, and land use change. It is based on several preceding peatland and soil maps of Indonesia, including the WI maps. Data sources include LREP data and RePPProt Land Form Classification maps [4], similar to WI maps [36]. In addition, data from several regional land and soil surveys provided by Haryono et al. [36] contributed to its development. MoA maps estimate the total peatland area of Indonesia to be 14,905,575 ha.

Page et al.’s [2] global assessment of tropical peatland area and carbon storage used another older map, the Land Form Classification Maps produced by the Regional Planning Program for Transmigration [4] for the extent and distribution of Indonesian peatlands. The program used a land system approach to classify Indonesian landforms and includes eight peatland categories. The maps indicate that Indonesia’s peatlands cover approximately 20,000,000 ha. These maps were produced from interpretation of aerial photography and Landsat satellite imagery in areas that lacked coverage. Although the landform maps imply “shallow” and “deep” peatlands, depth intervals are not provided, so these maps cannot be compared to the WI or MoA sources and are not included in the current analysis. In addition, the maps are less readily available and are used as a reference, rather than a national standard for spatial planning, management, or policy decisions regarding peatlands.

### Map comparisons

Direct detection of peat and continuous measurement of its thickness cannot be reliably accomplished using current remote sensing technology unless suitable vegetation proxies are available [41]. Much of Indonesia’s peatlands have undergone anthropogenic alteration or conversion, confounding peatland delineation by forest canopy characteristics or surface moisture content measured via satellite. Therefore, the WI and MoA maps rely on few, widely dispersed and geographically biased field measurements of peat presence and thickness. Each map reports peat depth as intervals, and depth classes are inconsistent. The WI map includes depth classes of 50–100, 100–200, 200–400, and 400–800 cm for Sumatran provinces [5]; <50, 50–100, 100–200, 200–400, 400–800 and 800–1200 cm for the Kalimantan provinces [6]; and <50, 50–100, 100–200 and 200–300 cm for Papuan provinces [7]. The MoA maps report standard peat depth intervals: 50–100, 100–200, 200–300, and >300 cm for all provinces [36]. We calculated peat area, volume, and carbon stores for each depth class, aggregated them across all depths, and compared results from each map source. Area, volume, and carbon storage of peat layers deeper than 200 cm were combined into a single category (>200 cm). To allow for comparison with the MoA map, the Irian Jaya Timur and Papua provinces reported in WI maps were merged, consistent with provincial boundaries established by the Indonesian government in 2003.

### Carbon storage

Peat carbon storage was calculated as:

$$ {\text{C}}_{\text{peat}} = {\text{V}}*{\text{C}}_{\text{d}} $$

where C_peat_ is carbon storage (kg C); V is peat volume (m^3^) and C_d_ is peat carbon density (kg C/m^3^), the product of bulk density (kg/m^3^) and carbon content (%C). When possible, regionally specific C_d_ values were used to calculate carbon storage by province. Carbon density data were compiled from peer reviewed literature and previous reviews (Table 1). Only data with %C values determined by elemental analysis using induction furnace methods were included. Carbon density calculated from default values or %C determined by semi-quantitative methods such as Walkely–Black wet combustion or loss on ignition (LOI) have been shown to be less reliable for highly organic soils [42, 43].

**Table 1:** Peat carbon density values (C_d_; kg C/m^3^) used for each province to calculate carbon stock

Province	C_d_	Sources
West Kalimantan	55.5	Neuzil [44], Anshari et al. [45], Warren et al. [15]
Central Kalimantan	61.6	Neuzil [44], Page et al. [46], Warren et al. [15]
South Kalimantan	61.6	Regional Central Kalimantan value used.
East Kalimantan	65.1	Average literature value
Aceh Darussalam	65.1	
Bangka Belitung	65.1	
Bengkulu	65.1	
Jambi	54.5	Warren et al. [15]
Lampung	65.1	Average literature value
Riau	69.8	Brady [47], Neuzil [44]
West Sumatra	65.1	Average literature value
South Sumatra	65.1	
North Sumatra	65.1	
Papua	65.1	
West Papua	65.1	

Average C_d_ from all listed sources was used for provinces absent from published literature

Since peat depth is reported on an interval scale, we calculated a low, medium, and high estimate for each map. Low estimates assumed all peatlands within each depth class were at the low end of the depth class. For example, a depth of 100 cm was used for all peatlands within the 100–200 cm class. Medium estimates assumed the depth of all peatlands within a given class were at the midpoint (i.e. 150 cm used for the 100–200 cm depth class), and high estimates assumed all peatlands within a given depth class were the highest end of the class (i.e. 200 cm used for the 100–200 cm interval). The WI maps also report peatlands <50 cm, therefore values of 10, 25 and 50 cm were used for low, medium and high volume estimates for that depth class. In the >200 cm depth class values of 300, 750, and 1000 were used for low, medium and high estimates respectively. The low estimate, 300 cm, is the upper end of depth reported by the MoA maps; the high estimate is the midpoint of the 800–1200 cm deepest depth class used in the WI maps (Kalimantan) and the medium estimate is the midpoint of the 300 cm minimum and 1200 cm maximum reported for the deepest depth class in the MoA and WI (Kalimantan) maps respectively. Best estimates of peat C storage were obtained by averaging the medium C stock estimates for each map.

## Results

### Peatland distribution and depth

The WI maps report 20,949,043 ha of peatlands distributed throughout four provinces in Kalimantan (Borneo), nine provinces in Sumatra, and three provinces in Papua (Table 2). Provinces containing the largest area of peatlands include Papua (27%), Riau, (19%) and Central Kalimantan (14%). The maps indicate that about 83% of Indonesian peatlands are less than 4 m deep. The distribution of peatlands in each depth class differs among Papua, Sumatra, and Kalimantan. Peatlands in Papua are assigned to shallower depths: 67% are within the 50–100 cm depth class, about 22% are within the 200–300 cm depth class, and no peatlands are mapped to be deeper than 300 cm. In contrast, 31% of peatlands in Sumatra are mapped >400 cm deep, 64% are within the 100–400 cm depth, and only about 5% are in the 50–100 cm depth class.

**Table 2:** Peat area (ha) by province and depth class published in the WI maps

Province	Depth classes						Total area	%
	<50 cm	50–100 cm	100–200 cm	200–400 cm	400–800 cm	800–1200 cm		
West Kalimantan	36,673	438,172	737,111	213,705	304,319		1,729,980	8
Central Kalimantan	75,990	958,486	462,399	574,978	661,093	277,694	3,010,640	14
South Kalimantan	76,785	79,368	78,766	96,710	–		331,629	2
East Kalimantan		264,559	112,511	219,703	100,224	–	696,997	3
Kalimantan total	189,448	1,740,585	1,390,787	1,105,096	1,065,636	277,694	5,769,246	28
Aceh	–	2219	175,558	96,274	–	–	274,051	1
Bangka Belitung	–	–	54,724	8896	–	–	63,620	0
Bengkulu	–	3588	37,317	20,082	2066	–	63,053	0
Jambi	–	92,520	207,621	316,305	100,392	–	716,838	3
Lampung	–	–	87,567	–	–	–	87,567	0
Riau	–	76,194	1,324,426	575,343	2,067,638	–	4,043,601	19
West Sumatra	–	89,353	42,817	22,199	55,865	–	210,234	1
South Sumatra	–	66,201	1,308,832	45,009	–	–	1,420,042	7
North Sumatra	–	47,212	228,424	49,700	–	–	325,336	2
Sumatra total	–	377,287	3,467,286	1,133,808	2,225,961	–	7,204,342	34
Papua	180,493	3,701,845	701,237	1,106,417	–	–	5,689,992	27
West Irian Jaya	–	844,442	–	129,775	–	–	974,217	5
East Irian Jaya	–	830,093	–	481,153	–	–	1,311,246	6
Papua total	180,493	5,376,380	701,237	1,717,345	–	–	7,975,455	38
Total	369,941	7,494,252	5,559,310	3,956,249	3,291,597	277,694	20,949,043	100
%	2	36	27	19	16	1	100	

For Papua provinces a 200–300 cm depth class is reported

The MoA maps indicate 14,905,575 ha of peatlands distributed throughout four provinces in Kalimantan, ten provinces in Sumatra, and two provinces in Papua (Table 3). Contrary to the WI maps, Sumatra contains the largest peatland area (43%), followed by Kalimantan (32%) and Papua (25%). The MoA maps indicate that about 80% of peatlands are <300 cm deep. Similar to the WI maps, peatland distribution across depth classes differs among islands. Consistent with WI maps, no peatlands are mapped to be >300 cm deep in Papua. In Sumatra, peatlands are distributed more evenly across size classes with 50–100, 100–200, and >300 cm depth classes containing 27% of the peatland area each, and the 200–300 cm depth class accounting for the remaining 19%. Peatlands in Kalimantan are also distributed more evenly, with the 50–100, 100–200, 200–300 and >300 cm depth classes containing 22, 29, 22, 27% of mapped peatlands, respectively.

**Table 3:** Peat Area (ha) by province and depth class published in the MoA maps

Province	Depth class				Total area	%
	50–100 cm	100–200 cm	200–300 cm	>300 cm		
West Kalimantan	421,697	818,460	192,988	246,989	1,680,134	11
Central Kalimantan	572,372	508,648	632,989	945,225	2,659,234	18
South Kalimantan	10,185	21,124	74,962	–	106,271	1
East Kalimantan	44,357	41,582	171,830	74,597	332,366	2
Kalimantan total	1,048,611	1,389,814	1,072,769	1,266,811	4,778,005	32
Aceh Darussalam	144,274	71,430	–	–	215,704	1
Bangka Belitung	42,568	–	–	–	42,568	0
Bengkulu	3856	802	2451	944	8053	0
Jambi	91,816	142,716	345,811	40,746	621,089	4
Kepulauan Riau	103	8083	–	–	8186	0
Lampung	49,331	–	–	–	49,331	0
Riau	509,209	908,553	838,538	1,611,114	3,867,414	26
West Sumatra	11,454	24,370	14,533	50,329	100,686	1
South Sumatra	705,357	515,400	41,627	–	1,262,384	8
North Sumatra	209,335	36,472	–	15,427	261,234	2
Sumatra total	1,767,303	1,707,826	1,242,960	1,718,560	6,436,649	43
Papua	1,506,913	817,651	319,874	1,718,560	2,644,438	18
Papua Barat	918,610	–	127,873	–	1,046,483	7
Papua total	2,425,523	817,651	447,747	–	3,690,921	25
Total	5,241,437	3,915,291	2,763,476	2,985,371	14,905,575	100
%	35	26	19	20	100	

There are 6,043,468 fewer hectares of peatland delineated on the MoA maps than on the WI maps (Table 4; Additional file 1: Tables 1–5). The MoA maps report less peatlands in all provinces except West Papua. However, the main difference (4,356,800 ha) between the MoA and WI maps is located in the Papua province, representing 71% of the total 6,115,734 fewer hectares of peatlands delineated across all provinces except West Papua. The second largest difference between maps is for Kalimantan provinces (991,241 ha), together accounting for 16% of the total difference, followed by Sumatran provinces (767,693), representing about 13% of the total difference between maps. Less peatland area on the MoA maps results from large reductions in deeper size classes which are not offset by gains in shallower size classes. For example in South Sumatra, there are 796,814 fewer hectares in depth classes >100 cm, and 639,156 more hectares in the 50–100 cm depth class on the MoA map, resulting in a net difference of 157,658 ha for the province. For depth classes >200 cm, net differences are negative for all provinces except Central Kalimantan where the MoA map indicates 64,449 ha more peatlands than the WI map. However, 462,104 ha fewer peatlands in depth classes <100 cm offset gains in classes >100 cm, resulting in 351,406 ha fewer peatlands in Central Kalimantan on the MoA map.

**Table 4:** Difference in peat area between MoA and WI maps (Area_MoA_–Area_WI_) by province and depth class

Province	Depth class				Total area	%
	<50 cm	50–100 cm	100–200 cm	>200 cm		
West Kalimantan	−36,673	−16,475	81,349	−78,047	−49,846	1
Central Kalimantan	−75,990	−386,114	46,249	64,449	−351,406	6
South Kalimantan	−76,785	−69,183	−57,642	−21,748	−225,358	4
East Kalimantan	–	−220,202	−70,929	−73,500	−364,631	6
Kalimantan total	−189,448	−691,974	−973	−108,846	−991,241	16
Aceh	–	142,055	−104,128	−96,274	−58,347	1
Bangka Belitung	–	42,568	−54,724	−8896	−21,052	0
Bengkulu	–	268	−36,515	−18,753	−55,000	1
Jambi	–	−704	−64,905	−30,140	−95,749	2
Lampung	–	49,331	−87,567	–	−38,236	1
Riau	–	433,118	−407,790	−193,329	−168,001	3
West Sumatra	–	−77,899	−18,447	−13,202	−109,548	2
South Sumatra	–	639,156	−793,432	−3382	−157,658	3
North Sumatra	–	162,123	−191,952	−34,273	−64,102	1
Sumatra total	–	1,390,016	−1,759,460	−398,249	−767,693	13
Papua	−180,493	−3,025,025	116,414	−1,267,696	−4,356,800	72
West Papua	–	74,168	–	−1902	72,266	1
Papua total	−180,493	−2,950,857	116,414	−1,269,598	−4,284,534	71
Total	−369,941	−2,302,146	−1,644,019	−1,776,693	−6,043,468	100
%	6	38	27	29	100	

Depth classes >200 cm were combined to allow comparison

### Peat volume

The smaller area of peatlands delineated on the MoA than on the WI maps, particularly in depth classes >200 cm, results in lower peat volume estimates (Table 5). Low, medium and high estimates of total peat volume for the WI maps are: 298, 476, and 643 km^3^, respectively, whereas for MoA maps they are 210, 391, and 519 km^3^, respectively (Additional file 1: Tables 6–10). Overall, the difference in medium peat volume estimates between maps is 85 km^3^. The largest difference is for Papua and is due to dissimilarities in peat area in depth classes 50–100 cm and >200 cm in Papua province. In total, Papuan provinces represent 62% of the peat volume difference between maps. Large gaps are also found for Sumatra (24 km^3^), especially for South Sumatra (7 km^3^), Jambi (5 km^3^) and Aceh Darussalem (3 km^3^). In Kalimantan, discrepancies between maps are mainly for East Kalimantan, where there is 5 km^3^ less peat volume on the MoA than on the WI maps.

**Table 5:** Medium estimates of peat volume (km^3^) calculated from MoA and (WI) maps, and differences between map sources

Island	Depth classes														
	<50 cm			50–100 cm			100–200 cm			>200 cm			Total		
	MoA	WI	Diff	MoA	WI	Diff	MoA	WI	Diff	MoA	WI	Diff	MoA	WI	Diff
Kalimantan	–	0.47	−0.47	7.86	13.05	−5.19	20.85	20.86	−0.01	121.83	124.86	−3.03	150.54	159.25	−8.71
Sumatra	–	–	0.00	13.25	2.83	10.43	25.62	52.01	−26.39	159.97	167.57	−7.61	198.84	222.41	−23.57
Papua	–	0.45	−0.45	18.19	40.32	−22.13	12.26	10.52	1.75	11.19	42.93	−31.74	41.65	94.23	−52.58
Total	–	0.92	−0.92	39.31	56.21	−16.90	58.73	83.39	−24.66	292.99	335.37	−42.38	391.03	475.89	−84.86

Detailed low, medium and high estimates of peat volume by province and depth class are provided in Additional file 1: Tables 6–10

### Peat carbon

Differences in peat carbon storage estimates between the MoA and WI maps reflect observed differences in peat volumes. Low, medium and high estimates of peat carbon storage for the WI maps are 19.23, 30.79 and 40.49 GtC, respectively (Table 6); and are 13.56, 25.33, and 33.77 GtC, for the MoA map, respectively (Table 7). The difference between medium peat carbon estimates is 5.46 GtC in total (Table 8; Additional file 1: Tables 11–19) and is mainly concentrated in Papua accounting for about 63% of the overall difference. In that province high discrepancies of 1.48 and 2.06 GtC are noticeable in the 50–100 cm and >200 cm depth classes. In Sumatra the difference between maps (1.49 GtC) is mostly located in South Sumatra (0.48 GtC), Jambi (0.26 GtC), and Aceh Darussalam (0.22 GtC). Finally, in Kalimantan most of the dissimilarity (0.55 GtC) relates to East Kalimantan (0.35 GtC).

**Table 6:** Low, medium and high estimates of peat carbon storage (GtC) by island and depth class, calculated using data from WI peat maps

Province	Depth class																							
	<50 cm			50–100 cm			100–200 cm			200–400 cm			400–800 cm			800–1200 cm			Total carbon (Gt)			%		
	Low	Med	High	Low	Med	High	Low	Med	High	Low	Med	High	Low	Med	High	Low	Med	High	Low	Med	High	Low	Med	High
Kalimantan	0.01	0.03	0.06	0.53	0.79	1.05	0.82	1.22	1.63	1.35	2.03	2.70	2.57	3.85	5.13	1.37	1.71	2.05	6.64	9.63	12.63	34.5	31.3	31.2
Sumatra	0	0	0	0.12	0.18	0.24	1.98	3.45	4.59	1.22	2.19	2.93	6.14	9.21	12.28	0	0	0	9.46	15.0.	20.04	49.2	48.8	49.5
Papua	0.01	0.03	0.06	1.75	2.63	3.50	0.46	0.68	0.91	0.91	2.79	3.35	0	0	0	0	0	0	3.13	6.13	7.83	16.3	19.9	19.3
Total	0.02	0.06	0.120.12	2.40	3.60	4.79	3.25	5.35	7.14	3.48	7.02	8.98	8.70	13.06	17.41	1.37	1.71	2.05	19.23	30.79	40.49			

Percentage (%) of each total C estimate is indicated in the last column. Detailed data by province are provided in Additional file 1: Tables 11–16

**Table 7:** Low, medium and high peat carbon storage (GtC) by island and depth class, calculated using data from MoA peat maps

**Province**	Depth class																	
	50–100			100–200			200–300			>300			Total carbon (Gt)			%		
	Low	Med	High	Low	Med	High	Low	Med	High	Low	Med	High	Low	Med	High	Low	Med	High
Kalimantan	0.31	0.47	0.62	0.81	1.21	1.62	1.32	1.64	2.30	2.30	5.76	7.68	4.74	9.08	12.22	34.8	35.9	36.2
Sumatra	0.58	0.87	1.16	1.14	1.71	2.28	1.62	2.03	3.57	3.57	8.92	11.90	6.91	13.53	18.91	50.8	53.4	56.0
Papua	0.79	1.18	1.58	0.53	0.80	1.06	0.58	0.73	0.00	0.00	0.00	0.00	1.90	2.71	2.64	14.0	10.7	7.8
Total	1.68	2.52	3.37	2.48	3.72	4.96	3.52	4.40	5.87	5.87	14.68	19.57	13.56	25.33	33.77			

Percentage (%) of each total C estimate is indicated in the last column. Detailed data by province are provided in Additional file 1: Tables 17–19

**Table 8:** Differences in medium peat carbon storage (GtC) between map sources (GtC_MoA_–GtC_WI_) by depth class and island

Province	Depth class					
	<50 cm	50–100 cm	100–200 cm	>200 cm	Total	%
Kalimantan	−0.03	−0.32	−0.01	−0.18	−0.55	10
Sumatra	0.00	0.69	−1.74	−0.45	−1.49	27
Papua	−0.03	−1.44	0.11	−2.07	−3.42	63
Total	−0.06	−1.07	−1.63	−2.70	−5.46	100

Depth classes >200 cm were combined to allow comparison, detailed data by province and for depth classes >200 cm are provided in Additional file 1: Tables 11–13

Depth classes >200 cm contain approximately 71 and 75% of Indonesia’s peat carbon according to the WI and MoA maps, respectively. MoA maps indicate that about 58% (14.68 GtC) of peat carbon is contained in peatlands >300 cm deep, and the remaining 42% (10.64 GtC) is in depth classes <300 cm. Similarly, 14.77 GtC are estimated in depth classes >400 cm on the WI maps; about 48% of the 30.79 GtC medium estimate. The 5.46 GtC difference in medium carbon storage estimates is distributed similarly across depth classes: there is a 2.76 GtC difference for depth classes <200 cm and a 2.70 GtC difference for depth classes >200 cm.

## Discussion

Indonesia’s peat C store is often cited as 57 GtC, the value reported by Page et al. [2] in their assessment of global tropical peat C pools. Similarly, Jaenicke et al. [13] estimated Indonesia’s peat carbon to be about 55 GtC. These results are in contrast with the 50 GtC stored in all tropical peatlands reported by Yu et al. [1]. The medium estimates of 25.3 and 30.8 GtC obtained from the MoA and WI maps (respectively) are substantially lower than those of Page et al. [2] and Jaenicke et al. [13] but are closer to the cumulative total of Wahyunto et al.’s [5–7] values available for the WI maps (37.2 GtC).

There is no current information to support or refute the validity of either map, as the accuracies of each map reflect the geographic bias of source data and field surveys, and neither map source provide quantitative estimates of uncertainty for peat volumes. Therefore we suggest a best estimate of Indonesian peat carbon of 28.1 GtC, the midpoint of the medium estimates of the two map sources. The broad range in carbon storage estimates are mainly caused by differences in peat volume among studies. Page et al. [2] estimated the peat volume to be 1138.2 km^3^, which was calculated assuming an average peat depth of 5.5 m across 206,950 km^2^ of peatland. Similarly, Jaenicke et al. [13] assumed an average peat depth of 4.5 m over 211,000 km^2^ of peatland, estimating the peat volume to be 949.5 km^3^. These peat volumes are about double our 475.89 km^3^ peat volume based on the midpoints of each depth interval multiplied by their spatial extent using WI maps (total area 209,490 km^2^).

Compared to national peat maps, the average peat depths used by Page et al. [2] and Jaenicke et al. [13] seem overestimated, as the average depth weighted to the upper bounds of each depth interval is about 3.1 m according to WI maps. A recent report by Hooijer and Vernimmen [48] suggested that both WI and MoA peat maps could consistently underestimate peat extent and peat thickness by 27 and 13%, respectively. However, these results must be interpreted cautiously because the data used to support them violate assumptions of randomness and independence. In their study, the authors compiled a secondary dataset of peat thickness measurements to assess the accuracy of existing WI and MoA maps. The data are in fact not random, as existing peat thickness measurements were automatically taken on known peatlands. Therefore areas mapped as peatlands that are not actually peat are not represented in the data. In addition, spatially-dependent data were treated as independent in the analysis. Large sample sizes of peat thickness measurements were taken from peatland areas that are deeper than indicated on the maps, thus skewing the number of measurements where peatlands are underestimated on maps, and the total average peat depth. Finally, the Hooijer and Vernimmen [48] report does not include Papua provinces, which comprise the largest uncertainties in peat volume and extent, as reported here. Nevertheless, it is clear from the existing field data evaluated by Hooijer and Vernimmen [48] that peat thickness is underestimated on both WI and MoA maps over wide areas where data are available. It is beyond the scope of this study to compare the accuracies of each map without a random, independent, nationwide dataset necessary to make the assessment. Rather, we provide the most rigorous estimate of peat C stocks using the best information currently available.

Overall, peat carbon estimates range from 13.6 GtC (the low MoA map estimate) to 40.5 GtC (the high WI map estimate). The wide range of these estimates reflects the current state of uncertainty in Indonesia’s peatland maps, both in areal extent and volume. The upper and lower bounds of the peatland carbon estimates are probably substantial over- and underestimates, as they assume all peatlands within a given depth interval are at the upper or lower value of the interval, and values represent the minimum and maximum spatial extent of peatlands included on the MoA and WI maps. It is important to note that our suggested value of 28.1 GtC is the midpoint of the two best estimates from each map source, each with a wide range of uncertainty. Additional field data are needed, particularly for Papua provinces, to better constrain the estimate of Indonesia’s total peat C store.

**Fig. 1:**
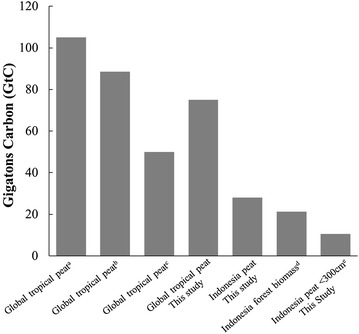
Comparative estimates of peat C pools and Indonesia’s forest biomass (^a^Dargie et al. [3]; ^b^Page et al. [2], ^c^Yu et al. [1]; Current study—global estimate replaces the 57 GtC value used by Page et al. [2] for Indonesia with 28.1 GtC estimated here, and accounts for additional peat C reported for the Central Congo Basin [3]); ^d^Saatchi et al. [50]—maximum estimate at 25% canopy threshold; and Indonesian peatlands <300 cm deep according to MoA maps

Our assessment of Indonesia’s peat carbon store revises the current global estimate of tropical peat carbon from 105 to 75 GtC (Fig. 1; [3]). The revised global estimate is almost the midpoint between the 50 GtC provided by Yu et al. [1], who used a carbon accumulation model and peatland distribution maps (Fig. 1), and 105 GtC suggested by Dargie et al. [3], who revised Page et al.’s [2] global estimate to include new data from the central Congo Basin. However, the peatland distribution map for Indonesia used by Yu et al. [1] was digitized from a descriptive figure provided by Page et al. [46], and is therefore a coarse representation of a large proportion of the total tropical peatland area. Using a refined modeling approach, Dommain et al. [9] quantified carbon stocks of West Indonesia (Sumatra and Kalimantan) at about 23 GtC, close to our 24.7 GtC medium estimate using the WI maps for the same area. The agreement between these values is expected, as Dommain et al. [9], used regionally specific values for carbon accumulation and the WI maps of peat distribution and depth in their analysis. Our carbon storage estimate for West Indonesia using the MoA maps is 22.6 GtC, remarkably similar to the 23 GtC reported by Dommain et al. [9]. However given the 1,758,934 ha difference in peat area between the MoA and WI maps for West Indonesia, it is likely that the peat volume modeled by Dommain et al. [9] was lower than the volumes obtained from WI maps for the same area, rather than suggesting higher accuracy of the MoA peat distribution maps.

Our medium carbon storage value for the WI maps is about 6.4 GtC lower than the total provided by Wahyunto et al. [5–7], despite using the same data for peat extent and depth. This difference is attributed to higher carbon density values used by Wahyunto et al. [5–7]. For example, C_d_ values used by Wahyunto et al. [5] for hemic and sapric peat in Sumatra were 82.5 and 125.6 kg C/m^3^, considerably higher than values typically found in the literature. The C_d_ values used by Wahyunto et al. [5–7] reflect the high values of representative bulk density for hemic and sapric peat, ranging from 170 to 280 kg/m^3^. Although carbon density of Indonesian peatlands varies considerably with bulk density and C content [15], we assumed C_d_ values ranging from 54.5 kg C/m^3^ for Jambi, Sumatra to 69.76 kg C/m^3^ for Riau, Sumatra based on published regional studies (Table 1). Where regional C_d_ values were unavailable, peatlands were assigned an average value of 65.1 kg C/m^3^. Although these values are higher than the assumed 50.4 kg C/m^3^ used by Page et al. [2], and 58.0 kg C/m^3^ used by Jaenicke et al. [13], C_d_ values assumed here are much lower than those used by Wahyunto et al. [5–7].

Indonesia’s peatland area is estimated to be between 14.9 Mha (MoA) and 20.9 Mha (WI). Overall differences in peatland area, volume and carbon storage between map sources are largely due to inconsistencies in the area and depth of peatland mapped in Papua province, which accounts for 71% of the difference in area, 62% of the difference in volume, and 63% of the difference in carbon storage. The expansive southern coastal plain of Papua contains one of the largest intact wetland forests in the world. Systematic survey of peat presence and its thickness is hindered by the logistical difficulties of accessing remote areas to acquire ground based data. Moreover, traversing the mosaic of mangrove, pandan marsh, Sago palm and swamp forests is costly, inefficient, and physically challenging. These factors preclude a rigorous systematic peatland survey of southern Papua, thereby introducing uncertainties in peatland maps. Although Papua accounts for the largest difference in peat volume and carbon storage, there are consistently fewer peatlands delineated on the MoA maps for all provinces except West Papua. A systematic revision of Indonesia’s peatland map based on reliable ground based data is necessary to assess peat volume and carbon storage with higher accuracy. Because of the inconsistencies between peat maps, they should be used for reference only. Intensive site-based surveys and peat evaluation at local or project levels are essential for spatial planning or land management purposes due to the uncertainties of peat presence and thickness at the project scale.

According to MoA maps, 80% of Indonesia’s peatlands are less than 300 cm deep and contain between 7.7 and 14.2 GtC, with a best estimate of 10.6 GtC, or 42% of Indonesia’s total peat carbon store. The WI maps indicate a similar percentage (83%) of peatlands mapped less than 400 cm deep, storing about 16.0 GtC. These estimates have significant implications for future carbon emissions and the fate of peatlands in shallower depth classes. According to Indonesian law (Presidential Decree 32/1990 and Government Regulation No 26/2008) land use conversion is allowable on peatland up to 300 cm deep. These laws stipulate that peat forests in the depth class >300 cm designated on the MoA maps are not to be drained and converted to other land uses and hydrological functions are to be retained. Therefore, assuming drainage persists to the base of the peat layer, 10.6 GtC are vulnerable to losses from land conversion, peat oxidation, and burning; equivalent to about 12 years of all global emissions from land use change at current rates (0.9 GtC year^−1^; [49]). This estimate is highly conservative, as it assumes the area of peatlands <300 cm is not underestimated, 100% compliance with environmental law, and does not consider biomass losses and ongoing losses from peatland >300 cm deep converted to other land uses prior to 1990. Furthermore, additional large-scale C losses resulting from uncontrolled peat burning or wildfire spreading into peat areas >300 cm deep are not included in the estimate. Additional losses could also occur if dry seasons are extended or are more severe due to climate change [50]. Although the calculation does not account for the preservation of peatlands <300 cm deep within protected areas, it is unlikely that the relatively small volume of protected shallow peat offsets carbon losses of converted or degraded peatlands >300 cm deep. In addition to large scale GHG emissions, the potential legal conversion of 80% of Indonesia’s peatland area—the proportion of peatland mapped <300 cm—is concerning considering the potential losses of biodiversity and associated ecosystem services. The potential loss of this carbon stock in “shallow” peat is equivalent to about half of all Indonesian forest biomass, further illustrating that accurate peat mapping, particularly of the 300 cm threshold and responsible peatland management is imperative to Indonesia’s efforts to reduce greenhouse gas emissions from the LULUCF sector.

## Conclusions

According to our assessment, Indonesia’s total peat carbon store lies somewhere between 13.6 and 40.5 GtC, with a best estimate of 28.1 GtC, the midpoint of medium carbon storage values calculated from WI and MoA maps. The large range of peat carbon estimates reflects considerable differences in peat volume provided by the map sources. The large uncertainties of peat extent, volume and carbon storage in Papua provinces, where data are most lacking, contribute to most of the differences in peat carbon storage estimates between map sources. Therefore, a robust peat survey of southern Papua province would help resolve the current discrepancies between peat maps.

Although lower than previous estimates, the 28.1 GtC approximation of Indonesia’s peatland carbon stocks presented here does not diminish the significance of Indonesia’s peat carbon storage at national and global scales (Fig. 1). Our estimate of peat carbon storage exceeds the 21.6 GtC maximum estimated carbon stock in above- and belowground biomass of all Indonesian forests by 30% [51], an observation similarly noted by Dommain et al. [9].

According to MoA national peat maps, approximately 80% of all Indonesian peatlands are less than 300 cm deep, and are thus allowable for conversion under current regulations. The past, ongoing and eventual conversion of these shallower peatlands could release approximately 10.6 GtC to the atmosphere assuming total peat loss, significantly contributing to global climate change [50]. Considering the high uncertainties in peatland extent, volume and carbon storage revealed in this assessment of current maps, a systematic revision of Indonesia’s peat maps to produce a single geospatial reference that is universally accepted would improve national peat carbon storage estimates and greatly benefit carbon cycle research, land use management and spatial planning.

## Additional file

**Additional file 1.** Warren et al. (2017) an appraisal of Indonesia’s immense peat carbon stock using national peatland maps: uncertainties and potential losses from conversion.
